# Effects of the Methanol Extract of *Basella alba* L (Basellaceae) on Steroid Production in Leydig Cells

**DOI:** 10.3390/ijms12010376

**Published:** 2011-01-14

**Authors:** Edouard Akono Nantia, Carine Travert, Faustin-Pascal T. Manfo, Serge Carreau, Thomas K. Monsees, Paul Fewou Moundipa

**Affiliations:** 1 Laboratory of Pharmacology and Toxicology, Department of Biochemistry, University of Yaoundé I, PO. Box 812 Yaounde, Cameroon; E-Mails: akonoed@yahoo.fr (E.A.N.); faustinpascal@yahoo.fr (F.-P.T.M.); 2 Biochemistry Laboratory-EA 2608-USC INRA 2006, IBFA, University of Caen, 14032, Caen, France; E-Mails: c.travert@uitcaen.unicaen.fr (C.T.); serge.carreau@unicaen.fr (S.C.); 3 Department of Medical Biosciences, University of the Western Cape, South Africa; E-Mail: thomas.monsees@tu-dresden.de

**Keywords:** aromatase mRNA, Basella alba, estradiol, rat Leydig cells, testosterone

## Abstract

In this study, Leydig cells were purified from 70 day-old Sprague Dawley male rats and incubated with 10 and 100 μg/mL of methanol extract of *Basella alba* (MEBa) for 4 hours followed by the evaluation of cell viability, steroid (testosterone and estradiol) production, and the level of aromatase mRNA. Results showed that MEBa did not affect Leydig cell viability. At the concentration of 10 μg/mL, MEBa significantly stimulated testosterone and estradiol production (p < 0.01 and p < 0.03, respectively), and enhanced aromatase mRNA level (p < 0.04). These observations suggest that MEBa directly stimulated testosterone, estradiol and aromatase mRNA levels in isolated Leydig cells.

## 1. Introduction

Plants have been used in the treatment of human illnesses for millennia, and many have shown a positive effect on male reproductive functions [1,2]. *Basella alba* is a plant used in traditional medicine in the West Cameroon region to treat sexual asthenia and infertility in man. Its methanol extract (MEBa) stimulated testosterone production in testicular fractions and Leydig cell cultures, and in normal adult albinos male rats [3–5]. Androgens are essential for male reproductive function and hypotestosteronemia and/or androgenic resistance are found in certain forms of male infertility [6]. In Leydig cells, testosterone is partly metabolized into estradiol by aromatase. This enzyme sustains the equilibrium between testicular testosterone and estradiol levels. Estradiol is also essential in the development and maintenance of male reproductive function with regard to animals deficient in the estrogen receptor gene or aromatase gene [7–9], and men with congenital aromatase deficiency [10]. Furthermore, the combined use of testosterone and estradiol in two hypogonadic patients with aromatase gene deficiency restored the serum hormone (testosterone, estradiol, LH and FSH) levels and the bone mineral density, while the single administration of testosterone or estradiol failed [9,10]. In addition, low levels of testosterone and estradiol (although the diminution of estradiol is less common than that of testosterone) are noted in age related hypogonadic individuals [11,12]. By stimulating testosterone production, MEBa may modulate androgen estrogen testicular homeostasis. This effect could contribute to the improvement of male reproductive function.

This work was thus aimed at determining whether MEBa could regulate androgen estrogen balance. To this end, freshly purified adult rat Leydig cells were incubated with MEBa, followed by determination of testosterone and estradiol release in the media, and cytochome P450 aromatase mRNA levels.

## 2. Results

### 2.1. Effect of MEBA on Leydig Cell Viability

**Figure 1:**
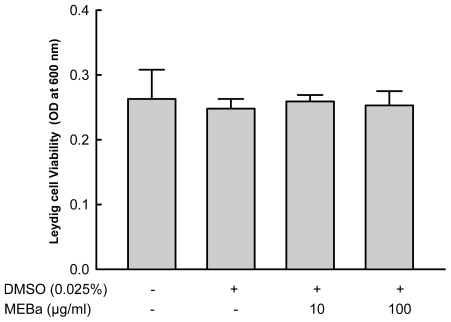
Viability of the Leydig cells treated with MEBa. Each value represents the mean ±SD of 3 experiments. Cells (3 10^5^/well, in duplicate) were incubated for 4 hours in the presence (+) or absence (−) of DMSO, or MEBa dissolved in DMSO. MEBa: methanol extract of *B. alba*.

As illustrated in Figure 1, the viability of Leydig cells was affected not by 10 or 100 μg/mL of MEBa, or the vehicle (0.025% DMSO).

### 2.2. Testosterone and Estradiol Production by Leydig Cells

The production of testosterone in control cells (basal) was 0.33 ± 0.05 ng/10^6^ 3β-HSD positive cells (Table 1). In the presence of 1 UI/mL and 10 UI/mL hCG, the production of testosterone increased 5-fold (p < 0.005) and 9-fold (p < 0.001), respectively. The concentration of 10 μg/mL MEBa significantly stimulated basal testosterone production (p < 0.01), and enhanced 1 UI/mL hCG-evoked testosterone level (p < 0.002).

As illustrated in Table 2, the basal estradiol production was 62.14 ± 8.68 pg/10^6^ 3 β-HSD positive cells. The estradiol level was increased 4-fold (p < 0.008) and 6-fold (p < 0.001) after incubation of cells with 1 UI/mL and 10 UI/mL hCG, respectively. The MEBa stimulated basal estradiol production (p < 0.03), and enhanced 1 UI/mL hCG evoked estradiol release at the concentration of 10 μg/mL (p < 0.002).

The concentration of 100 μg/mL of MEBa did neither affect testosterone nor estradiol release.

### 2.3. Aromatase mRNA Levels

**Figure 2:**
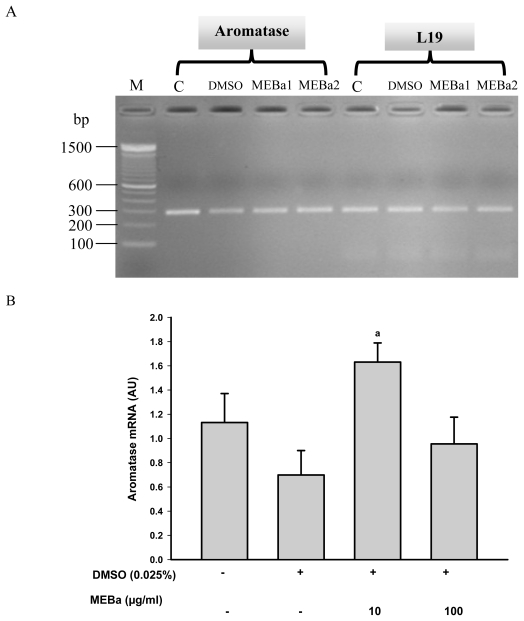
Aromatase transcripts **(A)** and normalized levels **(B)** in the Leydig cells. Aromatase transcripts visualization on a 2% agarose gel after RT-PCR. M corresponds to DNA ladder (100 bp ladder). Each value represents the mean ± SD of 3 experiments each done in duplicate. Cells (3 × 10^6^/well) were maintained for 4 hours in the incubation medium alone (**C**), or treated with MEBa (MEBa1: 10 μg/mL, MEBa2: 100 μg/mL) dissolved in 0.025% DMSO. Data are compared to those of Leydig cells treated with 0.025% DMSO. ^a^ p < 0.04 (Student Newman-Keuls test). MEBa: methanol extract of *B. alba.*

Aromatase mRNA levels (Figure 2A,B) were quantified and normalized against L19 mRNA as a reference. The mRNA levels of aromatase in the Leydig cells were significantly stimulated (p < 0.04) by 10 μg/mL of MEBa as compared to cells treated with 0.025% DMSO.

## 3. Discussion

Testosterone is essential for several physiological processes in man. It is involved in spermatogenesis, sexual arousal and virility, psychological well being, development and maintenance of the bone stature and erythropoïesis. Some biological effects of testosterone are mediated through its aromatization into estradiol [9,18–22]. The homeostasis between testosterone and estradiol is maintained by the enzymatic complex named aromatase located in the endoplasmic reticulum of the cells. Aromatase appears to be expressed in numerous tissues, and its activity is of great significance for the male reproductive function [10,23].

Moundipa *et al*. [4] showed that MEBa stimulates testosterone production by Leydig cells after 12 hours of incubation, with a maximum effect at 10 μg/mL. The androgenic activity of MEBa at 10 μg/mL was confirmed in our study after 4 hours of incubation. The testosterone- enhancing effect of this plant extract may be partly due the presence of terpenoid compounds [4], which may augment the pool of cholesterol—a substrate for steroid synthesis. However, the stimulatory effect of MEBa on testosterone was slightly reduced at the concentration 100 μg/mL. This suggests a desensitizing effect of the extract at high concentration, since Leydig cells viability was not affected.

In the presence of a suboptimal concentration of hCG (1 UI/mL), MEBa (10 μg/mL) enhanced both testosterone and estradiol production, but this stimulatory effect was annulled when a high concentration of hCG (10 UI/mL) was used. Human chorionic gonadotropin or LH binding to its receptor triggered the cAMP signaling cascade leading to rapid effects, including cholesterol mobilization*,* and elevated steroidogenic enzymes activity, and longer term transcriptional effects [23]. Active compounds of MEBa could optimize cellular pathways initiated by hCG through regulation of elements such as protein kinases and cAMP response elements.

The stimulatory effect of MEBa on estradiol level may result from its effect on aromatase gene transcription and translation into a biologically active enzyme. Indeed 10 μg/mL of MEBa increased the amount of aromatase transcripts, and this effect decreased when cells were incubated with 100 μg/mL of the extract. This observation is further corroborated by the stimulation of estradiol release, as well as the elevated level of its immediate precursor testosterone. In fact, estrogens positively regulate male reproductive function as shown by induction of spermatogenesis in the hypogonadal (hpg) mouse and the stimulation of spermatogonia cell proliferation in cryptorchid mice [25,26]. In addition, Asah [27] reported an increased sperm count, normal serum FSH, and fecundity in man suffering from oligospermia with small testes and elevated serum FSH after low dose estrogen testosterone combination therapy.

## 4. Materials and Methods

### 4.1. Plant Material and Preparation of the Methanol Extract of Basella alba

Fresh leaves of *B. alba* (identified at the Cameroonian National Herbarium as specimen N° 40720) were collected in Dschang (West Region of Cameroon) in August 2005, dried at room temperature and ground in powder. The methanol extract of *B. alba* was obtained through successive extraction in hexane, methylene chloride and methanol as previously described [3]. The extraction efficiency in methanol was 3%.

### 4.2. Chemicals

Dulbecco’s Modified Eagle’s Medium (DMEM), Ham-F12, Collagenase/ dispase, DNAse, Soybean Trypsin Inhibitor (STI) and NAD were purchased from Sigma Aldrich (France). RIA antibodies for testosterone and estradiol quantification were purchased from P.AR.I.S (Compiègne-France). Kits for the extraction of RNA and PCR were obtained from Promega (France). Other reagents were all of high quality grade.

### 4.3. Animals

Animals used in the experiment were 70 day-old Sprague–Dawley rats obtained from the animal house of the University of Caen, France. They were bred under standard conditions (12 h light:12 h darkness cycle and controlled room temperature) with standard rat food and water ad libitum. Their use was in accordance with the French Government Regulations (Services Vétérinaires de la Santé et de la Production Animale, Ministère de l’Agriculture) and approved by the Local Ethical Committee of the University of Caen.

### 4.4. Purification of Leydig Cells

Leydig cells were purified as described elsewhere [13]. Briefly, rats were sacrificed and testes dissected, removed and subjected to collagenase/dispase (0.05%) digestion in the presence of STI (0.005%) and DNase (0.001%) in DMEM/Ham F12 medium. The digested mixture was allowed to settle, the supernatant collected and centrifuged (900 rpm, 10 min, 18 °C). The resulting cell pellet was resuspended in the incubation medium and filtered through 30 nylon mesh membrane to obtain the crude interstitial cells. The Leydig cells were further purified on a discontinuous Percoll gradient (20–80%). After centrifugation of the gradient (2400 rpm, 20 min, 18 °C), Leydig cells fractions were collected and used for the assays. After purification, viable cells were counted using a hemocytometerbased trypan blue dye exclusion method and their purity was assessed by the 3β-hydroxysteroiddehydrogenase (3β-HSD) histochemical staining. The percentage of viable Leydig cells was more than 95% and their purity higher than 85%.

### 4.5. Study of the Effect of MEBA on Leydig Cell Viability

Purified Leydig cells (3 × 10^5^ cells/well/mL) were incubated for 4 hours (32 °C under 95% air/5% CO_2_) either with 0.025% DMSO (vehicle), 10 μg/mL and 100 μg/mL of MEBa. Plant extract concentrations were selected from previous studies conducted by Moundipa *et al*. [4]. After the incubation, culture plates were centrifuged (900 rpm, 10 min, 18 °C), supernatants discarded and cell pellets incubated with crystal violet solution (0.1% w/v in PBS 0.01 M, pH 7.4) for 30 min at 18 °C. The crystal violet was taken up by living cells during the incubation, and the excess dye eliminated through 3 washing steps with PBS. Diluted acetic acid solution (10%) was used to release the crystal violet taken up by cells, and the optical density of each well was determined at 600 nm using luminometer (BERTHOLD Technologies, Mithras LB 940) [14].

### 4.6. Study of the Effect of MEBA on Steroid Production and Aromatase mRNA Level

Leydig cells (3 × 10^6^ cells/well/2 mL) were treated either with vehicle (0.025% DMSO) or MEBa (10 or 100 μg/mL) or hCG (1 or 10 IU/mL). Human chorionic gonadotropin was used as a positive control to challenge Leydig cells and the concentrations used were after laboratory trials and from the previous study [15]. Plates were incubated (32 °C under 95% air/5% CO_2_) for 4 hours (time selected from preliminary study, which showed similar effect in steroid production after 4 hours or 12 hours of incubation). Culture plates were then centrifuged and supernatants used for the quantification of estradiol and testosterone levels by ^3^H-RIA using specific antibodies. Briefly, steroids were dissolved in phosphate albumin buffer (0.1 M, pH 7.3, containing 2% bovine serum albumin), incubated with radio-labeled testosterone or estradiol (3000 cpm/100 μL) and their respective antibody (Cross reactions between anti-estradiol and testosterone and vice-versa were always less than 0.01%). Unbound radio-labeled steroid was adsorbed to activated charcoal and separated by centrifugation. Antibody-bound radio-labeled steroid was thereafter determined using liquid scintillation counter. Estradiol was extracted from culture medium using diethylether (300:1, v/v) prior ^3^H-RIA quantification [15]. The inter- and intra-assay coefficients of variation were respectively 5 and 8% for testosterone, and 4 and 8% for estradiol. The detection limit was 3 pg and 4 pg per tube for estradiol and testosterone, respectively. Pellets containing cells were lysed with denaturing agent and RNA were recovered in an aqueous phase. RNA was further purified by precipitation with isopropanol and washing with cold ethanol [16]. Two μg of total RNA were further subjected to reverse transcriptase polymerase chain reaction (RT-PCR) in the medium containing 500 μM dNTP, 0.2 μg oligodT, 200 U MMLV-RT (Moloney murine leukeumia virus reverse transcriptase) as well as 20U RNasin (ribonuclease inhibitor) and primers [aromatase primers: 5_1555_-GCTTCTCATCGCAGAGTATCCGG-3 (sense), 5_1844_-CAAGGGTAAATTCATTGGGCTTGG-3 (antisense); L19 primers: 5_119_-GAAATCGCCAATGCCAACTC-3 (sense), 5_408_-ACCTTCAGGTACAGGCTGTG-3 (antisense)]. Five microliters RT-PCR products were then amplified in the presence of GoTaq Flexi DNA polymerase during 36 PCR cycles (for mRNA aromatase) or 24 PCR cycles (for mRNA L19, a DNA house-keeping gene [17] made up of denaturation at 95 °C (30 s), hybridization at 60 °C (30 s) and polymerization at 72 °C (45 s). Intensities of the obtained DNA products were determined after agarose gel electrophoresis using Image J software.

### 4.7. Statistical Analyses

Normality between data was checked by the Kolmogorov-Smirnov test, and differences between parameters of different treatments assessed by the Student Newman-Keuls’s test. Analyses were done using the Sigmastat 3.1 software (Systat Software Inc., San Jose, CA, USA).

## 5. Conclusions

MEBa stimulated estradiol production and aromatase mRNA by Leydig cells at 10 μg/mL. The traditional use of *B. alba* in the treatment of male infertility and sexual asthenia could be due to its capacity to stimulate not only androgens production, but also estrogens, thus maintaining the androgen estrogen balance necessary for normal male reproductive function. MEBa represents a semi-purified extract, and its further fractionation will enable isolation of pure active compound(s) exhibiting steroidogenic effect and male fertility enhancement.

## Figures and Tables

**Table 1:** Testosterone level of Leydig cells.

hCG	0 UI/mL	1 UI/mL	10 UI/mL
Control	0.33 ± 0.05		
0.025% DMSO	0.32 ± 0.01	1.63 ± 0.07^b^	2.81 ± 0.10^d^
MEBa 10 μg/mL	0.63 ± 0.04^a^	1.99 ± 0.09^c^	2.86 ± 0.17^d^
MEBa 100 μg/mL	0.35± 0.02	1.61± 0.05^b^	2.76 ± 0.08^d^

Data are expressed as ng/10^6^ cells. Each value represents the mean ± SD of 3 experiments each done in duplicate. Cells (3 × 10^6^/well) were maintained for 4 hours in the incubation medium alone (Control), or treated with MEBa dissolved in DMSO or with hCG. Data are compared to those of the Leydig cells treated with DMSO;

a p < 0.01;

b p < 0.005;

c p < 0.002;

d p < 0.001 (Student Newman-Keuls’s test). hCG: human chorionic gonadotrophin, MEBa: methanol extract of *B. alba*.

**Table 2:** Estradiol level of Leydig cells.

hCG	0 UI/mL	1 UI/mL	10 UI/mL
Control	62.14 ± 8.68		
0.025% DMSO	54.21 ± 11.50	201.37 ± 15.19^b^	331.44 ± 39.84^d^
MEBa 10 μg/mL	88.67 ± 6.05^a^	246.42 ± 21.67^c^	337.64 ± 35.31^d^
MEBa 100 μg/mL	69.26 ± 8.34	204.89 ± 28.38^b^	330.94 ± 42.17^d^

Data are expressed as pg/10^6^ cells. Each value represents the mean ±SD of 3 experiments each done in duplicate. Cells (3 × 10^6^/well) were maintained for 4 hours in the incubation medium alone (Control), or treated with MEBa dissolved in DMSO or with hCG. Data are compared to those of the Leydig cells treated with DMSO;

a p < 0.03;

b p < 0.008;

c p < 0.002;

d p < 0.001 (Student Newman-Keuls’s test). hCG: human chorionic gonadotrophin, MEBa: methanol extract of *B. alba*.
